# *Plasmodium falciparum* Gametocyte Density and Infectivity in Peripheral Blood and Skin Tissue of Naturally Infected Parasite Carriers in Burkina Faso

**DOI:** 10.1093/infdis/jiz680

**Published:** 2019-12-26

**Authors:** Elamaran Meibalan, Aissata Barry, Matthew P Gibbins, Shehu Awandu, Lisette Meerstein-Kessel, Fiona Achcar, Selina Bopp, Christopher Moxon, Amidou Diarra, Siaka Debe, Nicolas Ouédraogo, Ines Barry-Some, Emilie S Badoum, Traoré Fagnima, Kjerstin Lanke, Bronner P Gonçalves, John Bradley, Dyann Wirth, Chris Drakeley, Wamdaogo Moussa Guelbeogo, Alfred B Tiono, Matthias Marti, Teun Bousema

**Affiliations:** 1 Department of Immunology and Infectious Diseases, Harvard T.H. Chan School of Public Health, Boston, Massachusetts, USA; 2 Center for Excellence in Vascular Biology, Department of Pathology, Brigham and Women’s Hospital, Boston, Massachusetts, USA; 3 Centre National de Recherche et de Formation sur le Paludisme, Ouagadougou, Burkina Faso; 4 Radboud Institute for Health Sciences, Radboud University Medical Center, the Netherlands; 5 Wellcome Centre for Integrative Parasitology, Institute of Infection, Immunity and Inflammation, University of Glasgow, Glasgow, United Kingdom; 6 Centre Hospitalier Universitaire Régional de Ouahigoua, Université de Ouahigouya, Burkina Faso; 7 Immunology and Infection Department, London School of Hygiene and Tropical Medicine, London, United Kingdom; 8 MRC Tropical Epidemiology Group, London School of Hygiene and Tropical Medicine, London, United Kingdom

**Keywords:** *Plasmodium falciparum*, gametocyte, sequestration, transmission, elimination, anopheles

## Abstract

**Background:**

*Plasmodium falciparum* transmission depends on mature gametocytes that can be ingested by mosquitoes taking a blood meal on human skin. Although gametocyte skin sequestration has long been hypothesized as important contributor to efficient malaria transmission, this has never been formally tested.

**Methods:**

In naturally infected gametocyte carriers from Burkina Faso, we assessed infectivity to mosquitoes by direct skin feeding and membrane feeding. We directly quantified male and female gametocytes and asexual parasites in finger-prick and venous blood samples, skin biopsy samples, and in of mosquitoes that fed on venous blood or directly on skin. Gametocytes were visualized in skin tissue with confocal microscopy.

**Results:**

Although more mosquitoes became infected when feeding directly on skin then when feeding on venous blood (odds ratio, 2.01; 95% confidence interval, 1.21–3.33; *P* = .007), concentrations of gametocytes were not higher in the subdermal skin vasculature than in other blood compartments; only sparse gametocytes were observed in skin tissue.

**Discussion:**

Our data strongly suggest that there is no significant skin sequestration of *P. falciparum* gametocytes. Gametocyte densities in peripheral blood are thus informative for predicting onward transmission potential to mosquitoes and can be used to target and monitor malaria elimination initiatives.

## Teaser

*Plasmodium falciparum* gametocyte skin sequestration has long been hypothesized but never formally tested. We observed no evidence for higher gametocyte densities in skin tissue of naturally infected gametocyte carriers or blood meals of mosquitoes feeding on their skin.

Significant reductions in malaria burden in recent decades have stimulated malaria elimination initiatives [1], which may require approaches that specifically reduce malaria transmission [2]. Transmission of malaria depends on mature male and female gametocytes that circulate in the bloodstream and may be ingested by mosquitoes from the subdermal capillaries on blood feeding. Interestingly, mosquito infections with *Plasmodium falciparum* have been observed from gametocyte donors whose low gametocyte density seems incompatible with transmission [3], and mosquito infection rates are typically higher when mosquitoes feed directly on skin of gametocyte carriers, compared with feeding on venous blood through an artificial membrane [4, 5]. *P. falciparum* gametocyte aggregation [6] and sequestration could contribute to these observations by facilitating mosquito infections from low gametocyte densities. Skin sequestration has been observed for other human parasites, including *Trypanosoma brucei* [7], *Onchocerca volvulus, Mansonella*, *Leishmania infantum,* and *Leishmania donovani* [8–11], for which parasite burden in the skin is the best predictor of infectiousness.

Indirect evidence for skin sequestration of mature gametocytes in the microvasculature of the skin was first described in surveys conducted in the 1940s and 1950s in Belgian Congo (now Democratic Republic of Congo); gametocyte prevalence in a survey using skin scarification was 3-fold higher than in a survey 5 years earlier using finger-prick blood samples [12]. In a follow-up study with 1243 paired samples, a 13.4% increase in *P. falciparum* parasite prevalence and 15.6% increase in gametocyte prevalence was observed when blood and dermal fluids from skin scarification were used for sample preparation instead of finger-prick samples [13]. The hypothesized skin sequestration of intraerythrocytic *P. falciparum* gametocytes may be related to mechanical retention in cutaneous capillaries [14, 15], analogous to the reversible rigidity that is likely to prevent immature gametocytes from entering circulation [16, 17]. Alternatively, sequestration may be related to gametocyte cytoadhesive properties [18] mediated by parasite proteins on the infected red blood cell surface, analogous to adhesion of asexual *P. falciparum* parasites to receptors on human vascular endothelial cells by *P. falciparum* erythrocyte membrane 1 [19].

Although sequestration of mature gametocytes in the skin of naturally infected individuals remains speculative, it may play an important role in determining *Plasmodium* transmission efficiency [3, 15]. In the current article, we report on 2 independent studies in naturally infected gametocyte carriers from Burkina Faso, in which we quantified mature *P. falciparum* gametocytes in skin tissue, blood samples, and mosquito blood meals in association with onward transmission to *Anopheles* mosquitoes.

## METHODS

### Ethics Statement

Ethical approval was granted by the Ethical Review Committee of the Ministry of Health of Burkina Faso (deliberation nos. 2016-03-033 and 2017-02-018) and the Ethics Committee of the London School of Hygiene and Tropical Medicine (nos. 10489 and 11962). Individual written informed consent was obtained before enrollment. Malaria cases were treated according to national guidelines in Burkina Faso [20].

### Study Site and Population

Study participants were recruited in the village of Balonghin (Saponé district, Burkina Faso), where malaria transmission is seasonal and intense, with 51%–84% *P. falciparum* parasite prevalence and 49%–75% gametocyte prevalence by molecular methods [21].

### Study Design

#### Paired Skin Feeding and Membrane Feeding Study

This study was conducted in October-December 2017. Eligible participants (aged 15–50 years) were asymptomatic with *P. falciparum* monoinfection, with gametocyte densities of ≥1/500 leukocytes by microscopy (≥16/μL, assuming leukocyte counts of 8000/μL) and hemoglobin concentrations ≥8 g/dL (Table 1). Immediately after venipuncture using lithium heparin and ethylenediaminetetraacetic acid (EDTA) tubes (BD Vacutaine), 400–500 µL of heparinized blood in duplicate (for infectivity) and 400–500 µL of EDTA blood (for gametocyte quantification in blood meals) was offered to 60 starved 4–5-day-old female *Anopheles coluzzii* mosquitoes via an artificial membrane attached to a water-jacketed glass feeder maintained at 37°C [22]. After exactly 15 minutes of feeding in the dark, fully fed mosquitoes from heparin blood were transferred to storage cups and maintained for 6–8 days before dissection by 2 independent microscopists using 1% mercurochrome.

**Table 1.** Baseline Characteristics of Study Populations

Characteristic	Skin Biopsy Sampling (n = 9)	Paired Skin and Membrane Feeding Assays (n = 31)
Symptomatic status	Asymptomatic	Asymptomatic
Age, median (IQR), y	34.7 (30.7–36.4)	28.9 (18. 7–37.7)
Female sex, no. (%)	3 (33.3)	16 (51.6)
Hemoglobin, median (IQR), g/dL	11.8 (11.3–12.4)	14.2 (13.3–15.6)
Self-reported bed net use, no. (%)	9 (100)	25 (80.6)

Abbreviation: IQR, interquartile range.

From mosquitoes that fed on EDTA blood, 16 fully fed mosquitoes were killed after feeding for exactly 15 minutes, by sharp needle puncture of their midguts to release individual blood contents into 50 µL of RNAprotect Cell Reagent (Qiagen) for storage at −80°C. Direct skin feeding was performed immediately after membrane feeding. The participant’s calves were exposed to 60 mosquitoes that were allowed to feed for exactly 15 minutes. From this group, 12 fully fed mosquitoes were immediately killed, and their midguts punctured as described above. The remaining mosquitoes were maintained for dissection and oocyst screening, as described above. In addition to membrane and direct skin feeding assays, EDTA blood was collected by means of venipuncture (BD Vacutainer) and finger prick (BD Microtainer) and stored in RNAprotect Cell Reagent (Qiagen) at −80°C.

#### Skin Biopsy Study

In the period from September 2016 to March 2017, adults (aged 18–50 years) were invited to participate in the study if they had *P. falciparum* monoinfection with microscopy-detected gametocytes (as described above), hemoglobin levels ≥11 g/dL, and no skin infections or conditions and history of vasovagal responses to blood sampling or biopsies or allergy to lidocaine or prilocaine. Eligible individuals were asked to participate in sampling on 2 occasions, 4 days apart. At each occasion, skin biopsy samples including the dermis and hypodermis were taken from the lower part of the forearm (antebrachium; n = 2) and lower part of the calf (sura; n = 2) using single-use punchers (4-mm biopsy punch; Miltex). These locations are typically used for direct skin feeding experiments [23].

Procedures were performed by a qualified dermatologist 1 hour after application of a patch with a eutectic licodaine-prilocaine mixture as anesthetic (EMLA patch 5%; Aspen). Topical application was chosen to minimize vasoconstriction or dilution of tissue fluids that may occur on injection of anesthetics. Half of the biopsy samples (1 each from arm and leg) were immediately immersed in 2 mL of 10% formalin and maintained at 4°C overnight; after washing, samples were stored in 2 mL of 70% ethanol and stored at 4°C until further processing. Other biopsy samples were transferred to 1000 µL of RNALater stabilization reagent (Qiagen), incubated overnight at 2°C–8°C, and then transferred to −80°C. Finger-prick and venous blood samples were collected in EDTA-coated tubes, as described above.

### Molecular Analysis

Mosquito homogenates were pooled (4 mosquitoes in a total of 200 µL of RNAprotect Cell Reagent) with 4 pools (16 mosquitoes) for membrane feeding experiments and 3 for skin feeding experiment (12 mosquitoes). Nucleic acids from mosquito pools and from 100-μL venous and finger-prick whole-blood samples in RNAprotect Cell Reagent were isolated using a MagNAPure LC automatic extractor (Total Nucleic Acid Isolation Kit—High Performance, Roche Applied Science). Ring-stage asexual parasites, female gametocytes, and male gametocytes were quantified by means of individual quantitative reverse-transcription polymerase chain reaction (qRT-PCR) assays targeting *sbp1* [24], *Pfs25* [25], and *PfMGET* [26], respectively. RNA extraction from skin tissue was performed using the Qiagen RNeasy Plus Mini kit (Qiagen). In addition to qRT-PCR, the NanoString nCounter platform was used to quantify genes differentially expressed in specific *P. falciparum* parasite stages, as described in detail elsewhere [27] and in more detail in the Supplementary Methods.

### Histological Analysis of Skin Samples

Skin biopsy samples were processed as described in the Supplementary Methods. Sections were stained with mouse antibodies targeting CD31 (human endothelial cells) or rabbit antibodies targeting Pfs16 (gametocytes) [28] before the addition of goat anti-mouse immunoglobulin G–Alexa Fluor 488 (ThermoFisher, A-11029) or goat anti-rabbit immunoglobulin G–Alexa Fluor 647 (ThermoFisher, A-21245) secondary antibodies. Slides were viewed on a Nikon A1R inverted confocal microscope with a piezo z-drive to acquire z-stacks. In addition to skin biopsy samples, clots of cultured *P. falciparum* parasites (strains Pf2004, 3D7, and NF54) were generated to act as positive and negative controls [28]. Images and movies were generated using Image J software 2.0.0-rc-65/1.51w (this is a free ware from NIH).

### Sample Size Justification

For the paired skin feeding membrane feeding study, we assumed a mean of 15% infected mosquitoes in patent gametocyte carriers with a standard deviation of 20% and a within-subject correlation of the outcome of 0.5 [4, 29, 30]. If we then expected 2-fold higher mosquito infection rates in direct skin feeding, 17 paired membrane feeding and skin feeding experiments on patent gametocyte carriers would give 80% power to detect this difference at an α value of 0.05. Sample size justification for skin biopsy sampling was based on a paired comparison of the proportion of the total parasite population that is mature gametocytes.

We expected that 73% of the skin-snip biopsy samples had higher gametocyte concentrations, based on a meta-analysis that demonstrated enhanced infectivity after skin feeding than after venous blood membrane feeding [4]. Assuming that 70% of infected adults have detectable malaria parasites in skin tissue, allowing quantification of the proportion of parasites that are gametocytes, and with a lower limit of the 95% confidence interval >50%, 45 paired skin-snip samples and venous/finger-prick blood samples would give 83% power, with an α value of 0.05 to detect a different in parasite stage composition. A go/no-go criterion was defined, wherein an initial 10 gametocyte carriers were recruited for biopsy samples, and additional participants would be recruited only if gametocytes were detected in ≥50% of all samples.

### Statistical Analysis

All statistical analyses were performed using Stata software, version 15.0 (StataCorp). The proportions of infectious gametocyte carriers were compared between paired feeding experiments using the McNemar test, and the proportions of infected mosquitoes were compared between direct skin feeding and membrane feeding using logistic regression, controlling for study participant as a fixed effect. Spearman nonparametric correlation coefficients were calculated to assess associations between continuous variables, and the paired Wilcoxon rank sum test was used to compare parasite densities between blood or tissue samples from the same participants. The gametocyte fraction was calculated as the sum of male and female gametocytes, expressed as a proportion of the total parasite biomass of asexual ring-stage parasites and gametocytes.

## RESULTS

**Figure 1.**
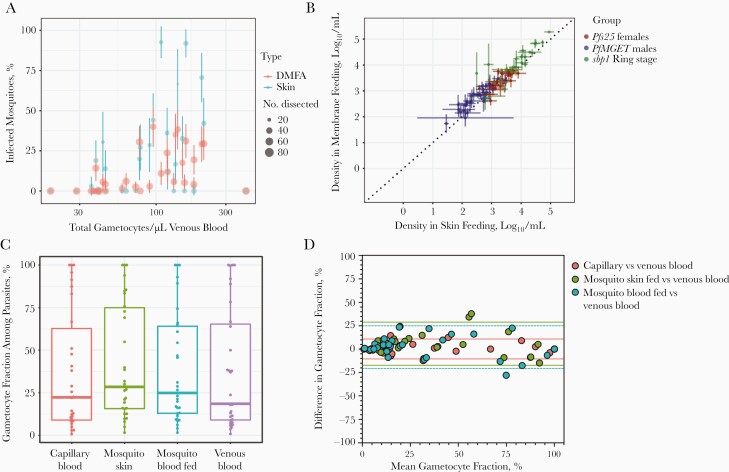
Density and infectivity of gametocytes in different blood compartments. *A,* Gametocyte density by quantitative reverse-transcription polymerase chain reaction (qRT-PCR) in venous blood in association with the proportion of mosquitoes that become infected when feeding directly on the skin of the blood donor (*blue*) or on venous blood offered through an artificial membrane feeder (*red*). Sizes of circles indicate the number of examined mosquitoes; error bars, the 95% confidence intervals around the proportions of infected mosquitoes. *B,* Density of ring-stage asexual parasites (*green*), male gametocytes (*blue*), and female gametocytes (*red*) by qRT-PCR in mosquito blood meals for direct feeding on the skin versus venous blood offered through an artificial membrane feeder. Error bars indicate the standard error of density estimates in pools of mosquitoes feeding directly on the skin (median, 3 pools) or on venous blood (median, 4 pools). *C,* Fraction of the total parasite biomass made up by gametocytes in finger-prick capillary blood samples (*red*), mosquitoes that fed directly on the skin (*green*), mosquitoes that fed on venous blood (*blue*) or venous blood samples (*purple*). Box plot depicts medians, interquartile ranges, and ranges, and dots represent individual samples. *D,* Bland-Altman plots (difference plots) for gametocyte fractions in different blood compartments. Red symbols indicate the difference in gametocyte fraction in capillary blood versus venous blood in relation to the mean fraction in these 2 compartments. Positive values indicate a higher gametocyte fraction in capillary blood compared with venous blood; dotted lines represent 95% limits of agreement. Green symbols represent agreement in gametocyte fraction measured in blood meals from mosquitoes that fed directly on skin tissue versus venous blood; blue symbols, agreement in blood meals from mosquitoes that fed on venous blood versus measurements directly in venous blood.

A total of 31 individuals aged 15–48 years (median, 29 years) participated in experiments with paired skin feeding and membrane feeding. The median number of dissected mosquitoes per experiment was 35 (interquartile range [IQR], 33–37) for direct skin feeding and 73 (69–82) for membrane feeding. Of 31 paired experiments, 18 (58.1%) direct skin feeding and 22 (71.0%) membrane feeding experiments resulted in ≥1 infected mosquito (*P* = .29). Total gametocyte density, quantified in venous blood by *Pfs25* and *Pfmget* qRT-PCR [26], was positively associated with the proportion of mosquitoes that became infected after direct skin feeding (ρ = 0.415; *P* = .02) or membrane feeding (ρ = 0.596; *P* < .001) (Figure 1A). The proportion of infected mosquitoes was higher with direct skin feeding than with membrane feeding assays (odds ratio, 2.01; 95% confidence interval, 1.21–3.33; *P* = .007). The medium number of oocysts was 4 (IQR, 2–7.5; maximum, 38) for mosquitoes that became infected after feeding directly on the skin and 2 (IQR, 1–5; maximum, 24) for those that became infected after feeding on venous blood through a membrane feeder.

To examine whether this higher infectivity in direct skin feeding assays was related to higher ingested gametocyte densities or to a higher gametocyte fraction in the blood meal, we directly quantified gametocytes and asexual parasites in mosquito blood meals. We quantified asexual parasites, by means of skeleton-binding protein 1 *sbp1* qRT-PCR [24], and gametocytes, by means of *Pfs25* and *Pfmget* qRT-PCR, in a median of 3 mosquito pools per participant (range, 2–3) from skin feeding and 4 pools per participant (range, 2–4) from membrane feeding mosquitoes; each pool contained 4 individual mosquitoes

We observed strong correlations between parasite quantities in pools of mosquitoes that fed on skin or venous blood through artificial membranes for asexual ring-stage parasites (*r* = 0.921; *P* < .001), male gametocytes *(r* = 0.790; *P* < .001), and female gametocytes (*r* = 0.655; *P* < .001) (Figure 1B). Opposite to our hypothesis, densities of asexual ring-stage parasites (*P* = .002) and female (*P* = .03) and male (*P* < .001) gametocytes were lower in blood meals taken directly from the skin compared with venous blood (Supplementary Figure 1). We also expressed gametocytes as fractions of the total parasite biomass. These fractions ranged from very low (<1% gametocytes in an individual with 21 086 ring-stage asexual parasites and 179 gametocytes per microliter) to 100% in 3 individuals without asexual parasites detected by qRT-PCR (Figure 1C). We observed no tendency toward a higher fraction of gametocytes in skin-fed mosquitoes or capillary blood compared with venous blood (Figure 1D) and a strong correlation between gametocyte fractions in the different compartments (Supplementary Figure 2).

**Figure 2.**
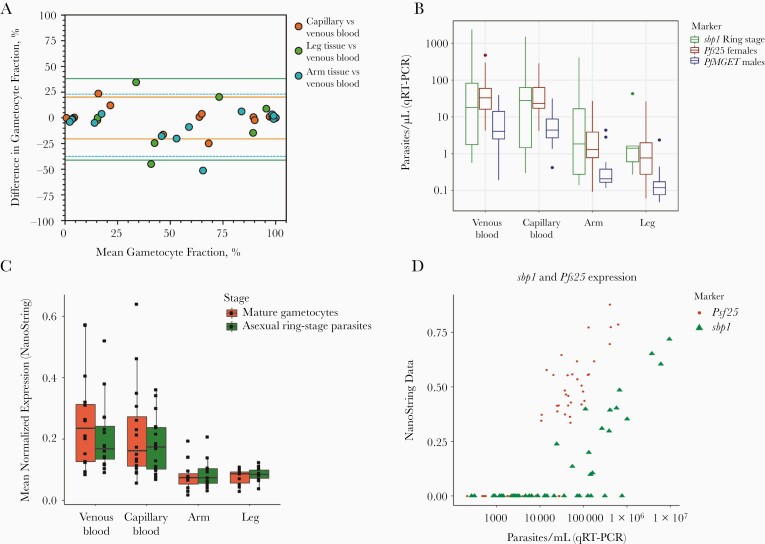
Quantitative reverse-transcription polymerase chain reaction (qRT-PCR) and NanoString comparison of parasite densities in skin biopsy samples and blood samples. *A,* Bland-Altman plots (difference plots) for gametocyte fractions in different tissue and blood compartments. Red symbols indicate the difference in gametocyte fraction in capillary blood versus venous blood in relation to the mean fractions in these 2 compartments. Positive values indicate a higher gametocyte fraction in capillary blood compared with blood; dotted lines represent 95% limits of agreement. Green symbols represent agreement in gametocyte fraction measured in tissue samples from the leg versus venous blood; blue symbols, agreement in gametocyte fraction in tissue samples from the arm versus measurements directly in venous blood. *B–D,* Relative numbers of asexual parasites and gametocytes in skin tissue from the arm, skin tissue from the leg, finger-prick and venous blood samples based on qRT-PCR (*B*) and NanoString data (*C*). NanoString data were normalized on the basis of background subtraction and expression of housekeeping genes. *D,* Correlation between estimates of ring-stage asexual parasites by *sbp1* and female gametocytes by *Pfs25* for qRT-PCR and NanoString data, showing good agreement but higher sensitivity for qRT-PCR.

In a complementary study, 9 adult gametocyte carriers participated in skin biopsy sampling. Male and female gametocytes and ring-stage asexual parasites were quantified by qRT-PCR to calculate the gametocyte fraction in finger-prick blood samples (16 observations in 9 donors), venous blood samples (16 observations in 9 donors), and skin tissue from the arm (13 observations in 7 donors) and leg (12 observations in 8 donors). Gametocytes were detected in all tissue and all blood samples by qRT-PCR; asexual parasites were detected in 17 of 25 tissue and in 30 of 32 blood samples. Gametocyte fractions were highly variable between donors (and between time points), whereas estimates from the different compartments from the same donor and time point showed strong correlation; the gametocyte fraction in venous blood was strongly associated with that in finger-prick blood (ρ = 0.947; *P* < .001), arm skin tissue (ρ = .928; *P* < .001) and leg skin tissue (ρ = 0.870; *P* < .001) samples, without any obvious bias toward higher gametocyte fractions in capillary blood or tissue samples compared with venous blood (Figure 2A).

Parasite density estimates per microliter of blood or tissue were generally lower in skin tissue than in blood samples (Figure 2B) and did not differ significantly between venous and finger-prick blood samples (*P* ≥ .12) or between leg and arm skin tissue samples (*P* ≥ .12). The same RNA aliquots were also processed for analysis by NanoString expression array, a highly sensitive probe-based expression platform that we have optimized for use in *P. falciparum* [31, 32]. With use of a previously defined stage-specific marker set for asexual rings and mature gametocytes [27, 32], there was no evidence for higher gametocyte transcripts in skin samples compared with blood samples (Figure 2C). The 2 approaches to quantify gene expression also showed a strong correlation for *sbp1* and *Pfs25* (Figure 2D).

**Figure 3.**
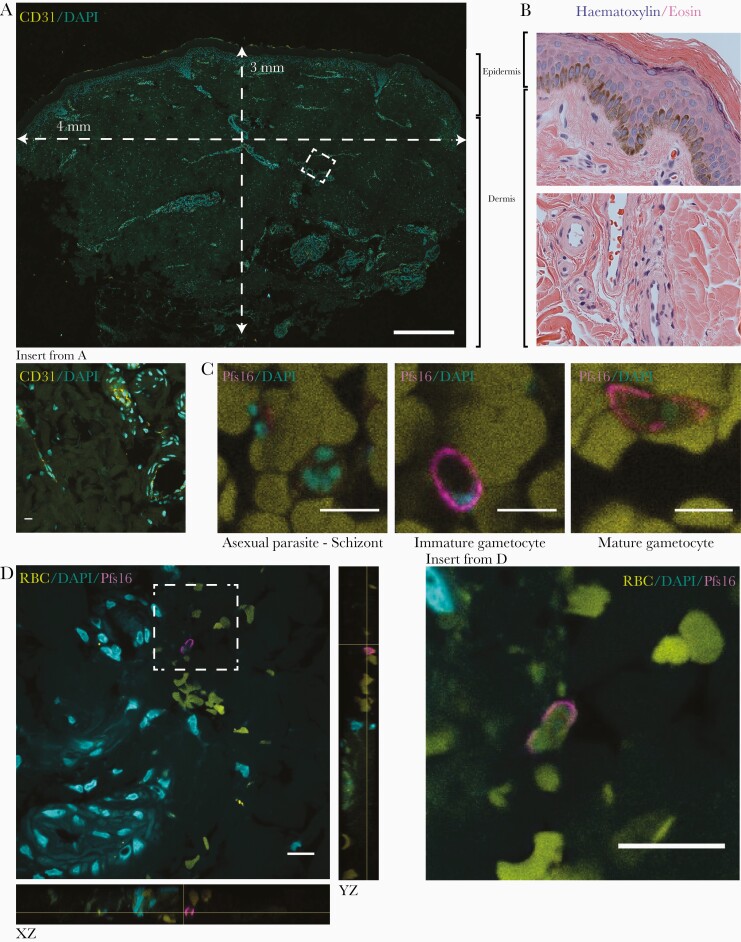
Histological analysis of skin samples. *A,* A 10-μm cross-section of a skin-snip sample from the leg, with dimensions indicated. The sample was stained with CD31 and 4’,6-diamidino-2-phenylindole (DAPI), and a maximum projection across the depth of the section is shown; insert represents a small section including several vessels stained with CD31. (Scale bar represents 500 μm; insert, 10 μm). *B,* A 3-μm section of a skin-snip sample from the arm, stained with hematoxylin-eosin. Sections in *A* and *B* show the different layers of the epidermis on top, followed by the dermis with multiple vessels. *C,* Samples were stained with DAPI (*cyan*) and Pfs16 (*magenta*) for gametocytes, with representative asexual parasite (*left*) and immature (*middle*) and mature (*right*) gametocyte images from control blood clots (scale bar indicates10 μm). *D,* Representative image of a gametocyte in skin samples from the arm. DAPI staining indicates several vessels in the vicinity of a gametocyte stained with Pfs16 (xz and yz orientations included to demonstrate the 3-dimensional nature of the tissue section and the gametocyte; scale bar indicates 10 μm). Abbreviation: RBC, red blood cell.

To directly detect gametocytes in subcutaneous tissue, we processed for imaging skin biopsy samples that were stored in formalin. Given the low densities of gametocytes predicted based on the qRT-PCR quantification (estimated median, 55.0 gametocytes [IQR, 28.2–153.0] in arm and 36.9 [11.6–98.3] in leg tissue samples), we established a protocol to image 10-μm sections with confocal microscopy, hence maximizing the detectability of sparse gametocytes (Figure 3A). Skin sections were initially analyzed using hematoxylin-eosin staining and labeled with the endothelial marker CD31 (Figure 3B) to confirm integrity of the tissue. Evaluation of gametocyte markers identified Pfs16 antibodies [28, 33] as highly specific and sensitive using the confocal imaging protocol (Figure 3C). Screening of ≥12 sections per skin-snip arm or leg sample from each participant identified several putative gametocytes. A Pfs16-positive cell with a characteristic crescent shape, 3-dimensional structure, and nuclear stain is shown in close association with a vessel (Figure 3D and Supplementary Movies 1 and 2). Based on these results, with low gametocyte detection rates by this highly sensitive fluorescence microscopy protocol, no further gametocyte carriers were recruited as tissue donors.

## Discussion

In the current study, we tested a long-standing hypothesis of *P. falciparum* gametocyte sequestration in skin tissue in 2 populations of naturally infected individuals in Burkina Faso. By combining mosquito feeding assays and direct quantification of parasite populations in skin tissue, mosquito blood meals, and blood compartments, we conclude that there is no evidence for significant skin sequestration of mature gametocytes.

Parasite sequestration in skin tissue is an intuitive explanation for how vector-borne parasites can maximize the likelihood of update by blood-feeding insects. This phenomenon, well demonstrated for a range of helminths [8–11] and protozoic trypanosomes [7], has remained speculative for *Plasmodium* parasites [15]. Two recent studies in Cameroonian parasite carriers that used microscopy as a diagnostic tool yielded conflicting results. One study observed higher *P. falciparum* parasite prevalence in finger-prick capillary blood samples than in venous blood samples from hospital patients [34]; the other found no differences for asexual parasites or gametocytes in gametocyte carriers [35]. The utility of finger-prick blood samples for estimating parasite biomass in skin tissue is uncertain.

Studies published in the 1940s and 1950s reported the superiority of skin scarification compared with finger-prick blood samples for parasite detection [12, 13, 36]. In the most extensive of these studies, in 1243 natural infections, 1 cm^2^ of skin of the scapular region was very slightly scarified with 4–5 light incisions, expressing a mixture of dermal fluids and capillary blood, with the first drop appearing richest in parasites [13]. This study demonstrated a 10%–20% increase in the prevalence of asexual parasites and gametocytes of *Plasmodium vivax, Plasmodium malariae,* and *P. falciparum,* but not *Plasmodium ovale.* Moreover, parasite density, expressed as parasites per 15 000 examined white blood cells, seemed to be increased [13]. In the current study, we therefore not only collected venous blood and finger-prick blood samples, but we also directly quantified parasite stage composition in skin tissue of naturally infected donors and in blood meals of mosquitoes that naturally fed on the skin of the corresponding donor.

We used a punch 4 mm in diameter to obtain a cylindrical core of tissue extending through the epidermis and down into the subcutaneous adipose tissue. This core represents an ideal tissue section for our purposes [15], as was taken from 2 location commonly used in direct skin feeding experiments [23]. We used the absolute quantity of gametocytes and the fraction of the total parasite biomass that is gametocytes as indicators of sequestration. In skin biopsy samples, we only sporadically encountered gametocytes by histology. We chose a fluorescence imaging protocol to image thick sections with confocal microscopy. This method allowed capturing of entire parasites and 3-dimensional reconstruction of parasite and surrounding tissues. Using Pfs16 labeling we classified gametocytes by crescent shape, 3-dimensional structure (as opposed to nonspecific speckles and autofluorescence, which is an inherent issue of this approach), nuclear stain, and presence of a surrounding red blood cell. The frequency of immunofluorescence-detected gametocytes in our tissue samples was lower than that shown by molecular methods in a tissue sample taken during the same visit. The quality of the skin tissue, tested by analyzing the tissue sections with hematoxylin-eosin staining, as well as by labeling for endothelial cells, clearly indicates that they were processed and preserved well.

In contrast, molecular detection of gametocytes was successful for all tissue samples with qRT-PCR and for the majority of samples with NanoString analysis. Because the volume of blood is unknown in tissue samples and gametocytes specifically are hypothesized to be enriched in skin tissue [12, 13, 15], we compared gametocyte fractions between different blood compartments and found no evidence for a higher gametocyte fraction in skin tissue. Gametocyte quantification in mosquito blood meals corroborated this finding and allowed a direct comparison of parasite densities. Again, we observed no evidence for higher concentrations of gametocytes in mosquitoes that fed directly on the skin of gametocyte donors compared with venous blood; estimated parasite densities were in fact higher in blood meals of mosquitoes that fed on venous blood. The reason for this is unclear and may be related to differences in mosquito blood meal volume. We observed a very strong association between gametocyte fractions from the different blood compartments. Although the sequestered parasite biomass may contain more mature parasite forms, our markers are specific of ring-stage parasites [24] or mature gametocytes with stable expression levels over time [26].

In the absence of clear evidence for skin sequestration, there must therefore be alternative explanations for the higher infection rates that we, in line with other studies [4, 5], observed in direct skin feeding experiments. Gametocyte activation may occur after phlebotomy and may reduce infection rates observed after membrane feeding. In addition, anticoagulants used in phlebotomy can have a pronounced effect on mosquito infection rates [37]. Although heparin is the preferred anticoagulant [37], it may still have a disadvantageous impact on sporogonic development. In malaria-naive individuals in whom *P. falciparum* gametocytes were induced during controlled human malaria infection studies, replacement of heparin plasma by serum resulted in increased mosquito infection rates [5]. Because human immune responses are unlikely to be of relevance in these gametocytemic volunteers, this observation provides additional indirect evidence for a transmission modulatory effect of heparin.

We observed no evidence for gametocyte sequestration in skin tissue. Our findings argue against a long-standing hypothesis that never had a proposed mechanism. Because the deformability of erythrocytes infected with mature gametocytes is similar to that of uninfected erythrocytes [16, 38] and there is no evidence for antigens on the surface of mature gametocyte-infected erythrocytes [39, 40], it is perhaps unsurprising that gametocyte concentrations are similar in the different blood compartments. Although direct skin feeding assays tend to result in higher infectivity compared with that observed in indirect feeding procedures using venous blood, our data demonstrate that any differences observed are based on technical rather than biological differences in the feeding procedure. Our findings also indicate that gametocyte levels in venous or finger-prick blood samples can be used to predict onward transmission potential to mosquitoes. Our findings thus pave the way for methods to quantify the human infectious reservoir based on conventional blood sampling approaches to support the deployment and monitoring of malaria elimination efforts for maximum public health impact.

## Supplementary Data

Supplementary materials are available at *The Journal of Infectious Diseases* online. Consisting of data provided by the authors to benefit the reader, the posted materials are not copyedited and are the sole responsibility of the authors, so questions or comments should be addressed to the corresponding author.

jiz680_suppl_Supplementary_Movie_1

jiz680_suppl_Supplementary_Movie_2

jiz680_suppl_Supplementary_Legends

## Notes

***Acknowledgments.*** We thank all study participants from Balonghin, Burkina Faso, for their participation. We also thank Fiona McMonagle for her guidance and assistance in the histology work.

Data underlying this article are available at https://datadryad.org/stash/share/_Di1z3S3jl2ahewKXHAXHfAtl7slSBGNAZmgueslqbI.

***Financial support.*** This work was supported by the European Research Council (fellowship ERC-2014-StG 639776 to T.B.), the Bill and Melinda Gates Foundation (grant INDIE OPP1173572), the Netherlands Organization for Scientific Research (VIDI fellowship 016.158.306 to T. B.), the National Institutes of Health (grant R21AI117304-01A1 to M. M.), the Royal Society (Wolfson Merit Award to M. M.), and the Radboud-Glasgow Collaboration Fund.

***Potential conflicts of interest.*** All authors: No reported conflicts. All authors have submitted the ICMJE Form for Disclosure of Potential Conflicts of Interest. Conflicts that the editors consider relevant to the content of the manuscript have been disclosed.
