# Supplementary material for: Plasmodium falciparum Gametocyte Density and Infectivity in Peripheral Blood and Skin Tissue of Naturally Infected Parasite Carriers in Burkina Faso
Source: J Infect Dis. 2019 Dec 26;223(10):1822–30. doi: 10.1093/infdis/jiz680 (PMC8161640; doi:10.1093/infdis/jiz680)
Supplement: jiz680_suppl_Supplementary_Legends [file jiz680_suppl_supplementary_legends.docx]

**SUPPLEMENTAL INFORMATION**

*P. falciparum* gametocyte density and infectivity in peripheral blood and skin tissue of naturally infected parasite carriers in Burkina Faso

**Supplemental methods**

**NanoString analysis**

The NanoString nCounter custom code set included differentially expressed genes to distinguish specific *P. falciparum* parasite stages as defined from our previous study [1]. A total of 456 parasite genes were included in the custom probe set including housekeeping genes. 161 genes representing asexual circulating stages, 147 genes representing asexual sequestering stages, 26 genes representing gametocyte rings, 27 immature gametocytes and 29 mature gametocyte genes. The remaining set was not annotated for any of these parasite stages. For NanoString analysis, 5 μl of purified total RNA was used for initial hybridization reaction. RNA from each sample was allowed to hybridize with reporter and capture probes at 65°C for 20 hours according to the nCounter gene expression assay protocol (NanoString Technologies). RNA-probe complexes were immobilized to nCounter cartridge followed by scanning in the nCounter Digital Analyzer. Data was first normalized by applying background subtraction and then normalized to expression of housekeeping genes using the R package ”NanoStringNorm”. The dataset was then quantile normalized using the R package ”aroma.light” and rank scaled. Mature gametocyte and asexual marker genes, as defined in [1], were then averaged per patient, per tissue and per visit.

**Histological analysis of skin samples**

Skin biopsies were processed by passing through an increasing alcohol gradient and xylene before embedded in paraffin wax. 10μm sections of biopsy samples were cut on a microtome and placed on adhesion slides (SuperFrost® Plus Gold, VWR). Slides were dried at room temperature for at least one hour then baked overnight at 42°C. The slides were allowed to reach room temperature before proceeding with the staining protocol. Slides were incubated at 60°C to melt the wax around the section; sections were cleared with xylene and rehydrated by passing through a decreasing alcohol gradient (xylene: 5 minutes twice; 100% ethanol: 3 minutes, twice; 90% ethanol: 3 minutes, twice; 70% ethanol: 3 minutes, twice). After incubation in distilled water for 3 minutes, heat induced antigen retrieval was performed using citrate buffer pH 6.0 (TCS Biosciences) in a table top autoclave. Slides were immersed in buffer using a metal rack in an empty tip box (without lid) and autoclave initiated until it reached 126°C, at which point the autoclave was unplugged and slides allowed to incubate in the autoclave for a further 10 minutes. Subsequently, the slides were removed and cooled in their buffer in a running water bath. Once at room temperature, slides were transferred to distilled water and then TBST (Tris Buffered Saline with 0.05% Tween 20) for 3 minutes each. Slides were then blocked with goat block containing 2.5% normal goat serum (Vector Laboratories) complemented with 2.5% normal human serum (ThermoFisher Scientific). All blocking and staining were performed in a humidified chamber. All staining solutions were removed by tapping the side of the slide gently on tissue paper. Excess liquid was removed by wicking away with tissue paper, being careful not to touch the sections. This was done to maintain intact, well-formed skin sections which are particularly delicate. After 30-60 minutes blocking at room temperature, the slides were incubated in primary antibodies diluted in goat block. Sections were stained with 1:20 (1.12μg/ml) mouse anti-CD31 (Cell Marque: clone JC70) at 4°C overnight or 1:1250 (1.04μg/ml) rabbit anti-Pfs16 [2] at room temperature for one hour. The slides were then washed with TBST for 3 minutes thrice before adding 1:100 goat anti-mouse IgG-AlexaFluor488 (ThermoFisher, A-11029) or 1:250 goat anti-rabbit IgG-AlexaFluor647 (ThermoFisher, A-21245) secondary antibody diluted in goat block and incubated at room temperature for 30 minutes. Following secondary antibody staining, the sections were washed twice with TBST and then once with TBS for 3 minutes each, before incubation with 2.5nM final concentration of DAPI diluted in TBS for 10 mins at room temperature. Sections were washed twice more in TBS for 3 minutes, before addition of TrueView autofluorescence quenching reagent (Vector Laboratories) and incubation for 3 minutes at room temperature. Sections were washed once more in TBS for 5 minutes before mounting with Vectashield Vibrance mountant (Vector Laboratories). Slides were viewed on a Nikon A1R inverted confocal microscope with Piezo Z-drive to acquire z-stacks. In addition to skin biopsies, clots of cultured *P. falciparum* parasites (strains Pf2004, 3D7 and NF54) were generated to act as positive and negative controls. Asexual and mixed asexual-immature gametocyte clots and mature gametocyte clots were generated as described previously [2]. Sections of formalin fixed paraffin embedded blocks were used to optimise Pfs16 antibody and DAPI staining and determine the staining of mature gametocytes. Using these controls gametocytes in the skin were determined by their circumferential staining with Pfs16 and obvious outline of a red blood cell. Red blood cells were determined by their bright autofluorescence under 488nm laser light. Images and movies were generated using Image J software.

**Supplemental Figure S1****. Bland-Altman plots (difference plots) for the density of parasites in mosquito blood meals.** Presented is the average parasite density in mosquito blood meals taken from skin tissue by direct skin feeding or venous blood by membrane feeding (X-axis) versus the difference between the two estimates (Y-axis). Positive values on the Y-axis indicate higher parasite densities in skin-fed mosquitoes; negative values indicate lower densities in skin-fed mosquitoes. Colours indicate ring-stage asexual parasites by *sbp1* qRT-PCR (green), female gametocytes by *Pfs25* qRT-PCR (red) and male gametocytes by *PfMGET* qRT-PCR (blue). Dashed lines indicate the mean difference and the 95% limits of agreement. For ring-stage parasites, there was strong evidence that correlation coefficient between the paired differences and means differed significantly from zero (Pitman’s Test of difference in variance, r = -0.923, p < 0.001); for male (p = 0.251) and female gametocytes (p = 0.559) this was not observed.

**Supplemental Figure S2. Correlation between gametocyte fraction in different blood compartments.** Presented is the fraction of the total parasite population that is gametocyte in venous blood (X-axis) versus on the Y-axis gametocyte fraction in finger prick capillary blood (red; Spearman ρ=0.970; p<0.0001), mosquitoes that fed directly on the skin (green; Spearman ρ= 0.916; p<0.0001), mosquitoes that fed on venous blood (green; Spearman ρ=0.912; p<0.0001).

**References**

1. Pelle KG, Oh K, Buchholz K, et al. Transcriptional profiling defines dynamics of parasite tissue sequestration during malaria infection. Genome Med **2015**; 7:19.

2. Joice R, Nilsson SK, Montgomery J, et al. Plasmodium falciparum transmission stages accumulate in the human bone marrow. Science translational medicine **2014**; 6:244re5.
